# Arterial stiffness but not carotid intima-media thickness progression precedes premature structural and functional cardiac damage in youth: A 7-year temporal and mediation longitudinal study

**DOI:** 10.1016/j.atherosclerosis.2023.117197

**Published:** 2023-08-04

**Authors:** Andrew O. Agbaje, Justin P. Zachariah, Tomi-Pekka Tuomainen

**Affiliations:** a Institute of Public Health and Clinical Nutrition, School of Medicine, University of Eastern Finland, Kuopio, Finland; b Children’s Health and Exercise Research Centre, Department of Public Health and Sports Sciences, Faculty of Health and Life Sciences, University of Exeter, Exeter, UK; c Section of Pediatric Cardiology, Department of Pediatrics, Texas Children’s Hospital, Baylor College of Medicine, Houston, TX, United States

**Keywords:** Left ventricular hypertrophy, Atherosclerosis, Metabolic syndrome, Left ventricular diastolic dysfunction, Pediatrics

## Abstract

**Background and aims:**

The longitudinal relations of cardiac indices with the aorta and carotid vessel and the time sequence for early cardiac disease development are uncharacterized in youth. We examined the temporal longitudinal associations of carotid-femoral pulse wave velocity (cfPWV) and carotid intima-media thickness (cIMT) with left ventricular hypertrophy (LVH) and diastolic dysfunction (LVDD).

**Methods:**

From the Avon Longitudinal Study of Parents and Children, UK birth cohort, 1856 adolescents (1011 females) at a mean (SD) age 17.7 (0.3) years were followed up for 7 years. Vicorder-measured cfPWV and ultrasound-measured cIMT were grouped in tertiles as low (reference), moderate, and high. Echocardiography measured cardiac abnormalities are left ventricular mass indexed for height^2.7^ (LVMI^2.7^) ≥51 g/m^2.7^ as LVH; relative wall thickness ≥44 as hiRWT; LVD function E/A <1.5 as LVD dysfunction (LVDD); and LV filling pressure E/e’ ≥8 as hiLVFP. Data were analysed with generalized logit mixed-effect models, cross-lagged path, and mediation structural equation models adjusting for cardiometabolic and lifestyle factors.

**Results:**

Over follow-up, LVH prevalence increased from 3.6% to 7.2% and LVDD from 11.1 to 16.3%. High cfPWV progression was associated with worsening LVH [Odds ratio 1.23 (1.13–1.35); *p* < 0.001] in the total cohort, males, overweight/obese, and normotensive. High cfPWV progression was associated with worsening hiLVFP in the total cohort, females, and normal weight. Likewise, high cIMT progression was associated with worsening LVH [1.27 (1.26–1.27); *p* < 0.0001] in the total cohort, overweight/obese and elevated BP/hypertensive. Neither cfPWV nor cIMT progression was associated with worsening hiRWT in the total cohort. In cross-lagged models, higher baseline cfPWV was associated with future LVMI^2.7^ (β = 0.06, SE, 5.14, *p* = 0.035), RWT, LVDF, and LVFP. However, baseline LVMI^2.7^, RWT, LVDF, and LVFP were not associated with follow-up cfPWV. Baseline cIMT was not associated with follow-up cardiac indices and *vice versa*. Cumulative increased systolic blood pressure (34.3% mediation) and insulin resistance (15.1% mediation) mediated the direct associations of cumulative cfPWV with cumulative LVMI^2.7^.

**Conclusions:**

Arterial stiffness progression temporally preceded worsening structural and functional cardiac damage in youth with increased systolic blood pressure and insulin resistance partly mediating the relationships. Future interventions aimed at attenuating premature cardiac damage in adolescents and young adults may consider a simultaneous treatment of both arterial stiffness, elevated blood pressure and insulin resistance.

## 1. Introduction

Atherosclerotic cardiovascular disease (ASCVD) and mortality remain a major public health concern with established evidence on the relationships of childhood through mid-adulthood cardiovascular risk factors with clinical events in later life [1–7]. Arterial stiffness predicts future ASCVD and causal ASCVD risk factors such as hypertension, insulin resistance, obesity, cardiac damage, kidney damage, and type 2 diabetes mellitus in the young and middle-aged population [4,8–14]. Arterial stiffness and carotid thickness are utilized as surrogates of ASCVD risk factor accumulation in the paediatric population, but their respective prospective associations to future outcomes like ventricular remodelling independent of the correlated ASCVD risk factors are unknown [4,13,15–18]. Putative mechanisms link stiffer blood vessels to higher ventricle-distending wall stress and then compensatory distending stress-normalizing wall thickening and stiffening, termed ventricular-vascular interactions [3,19–21]. Among adults, it is well established that left ventricular hypertrophy (LVH), LV diastolic dysfunction (LVDD), and arterial stiffening are markers of future ASCVD [3,19,21]. Paediatric blood pressure (BP) and obesity have been prospectively associated with ventricular remodelling measured once in middle age [5–7], but longitudinal relations between LV structure and function and early life arterial indices remain uncharacterized [22,23].

From a public health and clinical perspective, whether early life arterial stiffness and carotid thickness independent of BP and obesity are potential risk factors for LVH and LVDD would signal the earliest time point to prevent ventricular remodelling and consequent ASCVD including heart failure with preserved ejection fraction, specifically in adolescents and young adults. Previously, approaching causal inference by defining the time sequence for the development of structural and functional cardiac damage in relation to progression in youth arterial stiffening was hampered by the lack of repeated cardiac and arterial measures. Moreover, there is no prospective evidence on the traditional risk factor cumulative determinants of repeated measures of altered cardiac structure and function in youth. The pathway through which arterial stiffness may exert its influence on cardiac structure and function in youth is unclear.

We examined the temporal causal path and mediation longitudinal associations of progression in arterial stiffness and carotid thickness with the risk of worsening structural and functional cardiac damage among adolescents and young adults, using data from the Avon Longitudinal Study of Parents and Children (ALSPAC) birth cohort, England, United Kingdom. We hypothesized 1) that arterial stiffness and carotid thickness progression are associated with the risk of worsening LVH and other cardiac functional damage indices progression even after adjustment for BP and obesity 2) that worsening arterial structure and function temporally preceded worsening cardiac damage and 3) that traditional risk factors such as BP, insulin resistance, and body composition mediate the longitudinal associations of vascular and cardiac indices.

## 2. Patients and methods

### 2.1. Study cohort

Details of the ALSPAC birth cohort have been published earlier [8, 24–26]. The ALSPAC birth cohort investigates factors that influence childhood development and growth. Altogether, 14,541 pregnancies from women residing in Avon, southwestern England, UK, who had a total of 14,676 foetuses, were enrolled between April 1, 1991, and December 31, 1992. When the oldest children were approximately 7 years of age, an attempt was made to bolster the initial sample with eligible cases who had failed to join the study originally resulting in 913 additional pregnancies. The total sample size for analyses using any data collected after 7 years of age was 15,454 pregnancies, resulting in 15, 589 foetuses. Of these 14,901 were alive at 1 year of age. Regular clinic visits of the children commenced at 7 years of age and are still ongoing. Study data at 24 years were collected and managed using REDCap electronic data capture tools [27]. For our analysis, we included participants who had complete cfPWV, cIMT, LV mass indexed for height (LVMI^2.7^), and relative wall thickness (RWT) measurements at baseline, age 17 years clinic visit (Supplemental Fig. 1). Ethical approval for the study was obtained from the ALSPAC Ethics and Law Committee and the Local Research Ethics Committees. Consent for biological samples has been collected in accordance with the Human Tissue Act (2004). Informed consent for the use of data collected via questionnaires and clinics was obtained from participants following the recommendations of the ALSPAC Ethics and Law Committee at the time.

### 2.2. Anthropometric, cardiometabolic, and lifestyle factors measures

Anthropometry (height and weight) of participants at ages 17 and 24 years was assessed by observing standard protocols and body mass index (BMI) was computed as weight in kilograms per height in meters squared. Overweight/obesity was categorized as BMI ≥24.99. Heart rate and systolic and diastolic BP were measured at ages 17 and 24 years using an Omron 705-IT as previously detailed [8,9]. Participant’s with ≥130 mmHg systolic BP were categorized as elevated systolic BP/hypertension. Fasting blood samples at ages 17 and 24 years were collected, spun, and frozen at −80 °C and later assayed for high-density lipoprotein cholesterol, low-density lipoprotein cholesterol, triglyceride, glucose, insulin, and high-sensitivity C reactive protein as detailed previously [8,9,28]. Homeostatic model assessment for insulin resistance was computed from (fasting insulin x fasting glucose/22.5) [29, 30]. Total fat mass and lean mass were assessed using a dual-energy X-ray absorptiometry scanner at 17-year and 24-year clinic visits [31]. Questionnaires to assess smoking behaviour were administered at the 17-year and 24-year clinic visits. At the 17-year clinic visit, participants were briefly asked about their personal and family (mother, father, and siblings) medical history such as a history of hypertension, diabetes, high cholesterol, and vascular disease. The participant’s mother’s socioeconomic status was grouped according to the 1991 British Office of Population and Census Statistics classification [15]. Sedentary time, light physical activity, and moderate to vigorous physical activity at the age 15 years were assessed with ActiGraphTM accelerometer worn for 7 days but ActiGraph GT3X + accelerometer device worn for four consecutive days at age 24 years clinic visit, ideally starting the day after the clinic visit [9,32,33].

### 2.3. Vascular measures

At both time points, cfPWV was assessed from pressure waveforms obtained using the Vicorder device (Skidmore Medical, Bristol, UK) which has two BP measurement channels and two Velcro pressure sensor cuffs [8,9]. A cuff was placed over the right carotid artery in the participant’s neck, while another was located over the femoral artery in their upper right thigh. The distance between the participant’s suprasternal notch and the top of the thigh cuff was measured, as was the distance between their suprasternal notch and the bottom of the neck cuff on the right side. Transit time to the nearest 0.01 ms and cfPWV were automatically computed from measurements of pulse transit time and distance travelled by the pulse between two recording sites. All measurements were taken independently by one of two trained vascular technicians (inter-observer mean difference 0⋅2 m/s, SD 0⋅1) [8,9]. The oscillometric measure of cfPWV has been validated in adolescents [34].

At baseline, cIMT was assessed by ultrasound using a linear 12-MHz transducer (Vivid7, GE Medical, Chicago, Illinois) and the maximum cIMT value of 3 serial measures at 3 different cardiac cycles was used in the study. Interobserver variability for cIMT was assessed in a separate sample of 25 young adults (coefficient of variation: 4.4 ± 2.2%) [8,9]. The right and left common carotid arteries at the age 24 years were imaged using an ultrasound machine (CardioHealth Panasonic and a 13.5 MHz linear array broadband transducer (probe; centre frequency 9.0 MHz) in line with standard protocols [8,9]. Participants were placed in a supine position with the head rotated by 45^◦^ from the midpoint. An automated guide line was placed at the bulb (a longitudinal scan that included the common carotid artery and the carotid bifurcation) with the region-of-interest box and IMT trace lines automatically positioned 1 cm away from the guide line. The scanner automatically saved an image when the region-of-interest box turned green, indicating good image quality. An automated cIMT measurement, recorded from the posterior wall of the artery, was saved after three consecutive cardiac cycles. When interrogating the common carotid, the CardioHealth system calculated and displayed the cIMT that is updated at each detected R-wave of the cardiac cycle. Once the measurement achieved a predefined quality threshold, scanning automatically stopped and a report was generated. Raw data were checked for outliers and cIMT value > 1.0 mm was reviewed by a trained research scientist to assess validity. Abnormal values due to measurement error were removed. Participants had between 1 and 3 cIMT measures for each of the right and left carotid arteries. For the present analysis, the measurement of the right and left common carotid arteries was averaged as cIMT [8,9]. cfPWV and cIMT were categorized in tertiles as low (reference), moderate, and high tertile categories.

### 2.4. Cardiac structure and function measures

At 17 years, echocardiography was performed according to American Society of Echocardiography guidelines [35,36] by 1 of 2 experienced echocardiographers using an HDI 5000 ultrasound machine (Phillips Healthcare, Amsterdam, The Netherlands) equipped with a P4–2 Phased Array ultrasound transducer. At 24 years, echocardiography was performed by two experienced echocardiographers using a Philips EPIQ 7G Ultrasound System equipped with a X5–1 transducer. Philips Q-station was used for the M-mode, 2D, and Doppler echo analyses, while TomTec software was used for the 3D echo analyses. Measures of cardiac structure were LVMI^2.7^ to allometrically scale to ideal body mass [32,37] and RWT computed from septal wall thickness, posterior wall thickness, and left ventricular (LV) diastolic diameter. Measures of cardiac function were LVD function E/A wave ratio (LVDF) and LV filling pressure E/e’ wave ratio (LVFP). Pulsed Doppler examination of transmitral flow was recorded from the apical four-chamber view. For LV measurements the sample volume was positioned between the mitral annulus and the tips of the mitral leaflets with the position adjusted to maintain the sample volume at an angle as near parallel to transmitral flow as possible with the participant in passive end-expiration. The peak flow velocity of the early (E) and atrial (A) waves were measured from the three consecutive cardiac cycles displaying the highest measurable velocity profiles. Similar measurement (e’) was conducted at the tricuspid valve. Tissue Doppler echocardiography was performed in the 4-chamber view on the lateral, inferior, and septal LV walls to obtain myocardial wall velocities. Data were acquired with the beam parallel to the wall of interest and with optimal settings to ensure no over-gain of the low-velocity signals. A 5 mm sample volume was placed at the level of the mitral valve annulus and a loop of 8–10 cardiac cycles was recorded. The reproducibility of echocardiographic examinations was assessed by recalling 30 participants and repeating their measurements. The intraclass correlation of repeated measurements ranged from 0.75 to 0.93 (intraobserver) and 0.78 to 0.93 (interobserver) [38]. LV hypertrophy (LVH) was defined as LVMI^2.7^ ≥51 g/m^2.7^ since it predicts hard cardiovascular events, increased RWT (hiRWT) as ≥0.44, LVD dysfunction (LVDD) as E/A <1.5 and increased LVFP (hiLVFP) as E/e’ ≥8 [39–41].

### 2.5. Statistical analysis

Participant’s descriptive characteristics were summarized as means and standard deviation, medians and interquartile ranges, or frequencies and percentages. We explored sex differences using independent t-tests, Mann Whitney-U tests, or Chi-square tests for normally distributed, skewed or dichotomous variables, respectively. We assessed the normality of variables and logarithmically or reciprocally transformed skewed variables prior to further analyses. We examined the cumulative cardiometabolic and lifestyle risk factors determinant of cardiac structure and function progression using the linear mixed-effect model. The covariates were sex, family history of hypertension/diabetes/high cholesterol/vascular disease, social-economic status, and repeated measures of age, low-density lipoprotein cholesterol, insulin, triglyceride, high-sensitivity C reactive protein, high-density lipoprotein cholesterol, heart rate, glucose, systolic BP, fat mass, lean mass, smoking status, and moderate to vigorous physical activity, light physical activity and sedentary time.

Structural equation modeling with autoregressive cross-lagged path design was used to examine the separate temporal path associations of cfPWV and cIMT (vascular indices) with each of LVMI^2.7^, RWT, LVDF, and LVFP (cardiac indices). The cross-lagged models first tested the separate associations of cfPWV and cIMT at 17 years with each of LVMI^2.7^, RWT, LVDF, and LVFP at 24 years; and secondly tested the separate associations of LVMI^2.7^, RWT, LVDF, and LVFP at 17 years with cfPWV and cIMT at 24 years. These models were adjusted for statistically significant determinants of each marker of cardiac structure and function progression. In the cross-lagged design, the potential association could be vascular index progression toward cardiac index progression, cardiac indices toward vascular indices or bidirectional associations between vascular and cardiac indices. If a path from cfPWV and cIMT at time t-1 (17 years) to each of LVMI^2.7^, RWT, LVDF, and LVFP at time t-2 (24 years) reached significance (p-value<0.05), changes in the earlier variables are considered to lead to changes in the later one, and *vice versa*. A stronger predictive effect is determined by a larger standardized regression coefficient. We concluded that the cross-lagged models had good fit with the following indices: the root-mean-square error of approximation (<0.0001, the value < 0.05 is considered to indicate a good model-data fit), the normed fit index (>1.000), the relative fit index (>0.992), the incremental fit index (>1.000), the Tucker–Lewis Fit Index (>1.012), the comparative fit index (>1.000), which are considered good fit if values are >0.90. We included error terms in the model.

We examined the separate association of tertile categories of cfPWV and cIMT progression with each of the risks of worsening LVH, hiRWT, LVDD, and hiLVFP using generalized mixed-effect models with logit link. Analyses were adjusted for all above-listed covariates measured at both baseline and follow-up, including follow-up time. The effect estimates of the time by predictor interaction explained the impact of cfPWV and cIMT progression on the risk of worsening cardiac indices. Random intercept at the participant level was included in all models. We define LVH progression as the worsening of LVMI at follow-up in participants with LVH at baseline. All covariates were selected based on previous studies [4,8–10,39,42,43]. We presented sex-stratified results, body mass index-weight stratified results, and elevated BP/hypertension stratified results in the supplementary appendix. For clinical reference, [16] normative percentile values of vascular and cardiac structure and function measures are presented in Supplemental Table 1.

Lastly, the mediating path analyses using structural equation models separately examined the mediating role of cumulative total body fat mass, lean mass, systolic BP, heart rate and insulin resistance on the associations of cumulative cfPWV with each of cumulative LVMI^2.7^, RWT, LVDF, and LVFP from ages 17 through 24 years. Analyses were adjusted for sex, age, the time between exposure and outcome measure, high sensitivity C-reactive protein, heart rate, systolic BP, smoking status, family history of hypertension/diabetes/high cholesterol/vascular disease, socioeconomic status, high-density lipoprotein cholesterol, low-density lipoprotein cholesterol, triglyceride, lean mass or total fat mass, glucose and insulin, sedentary time, light physical activity, moderate to vigorous physical activity depending on the mediator. β from the mediation analyses are standardized regression coefficients. The path models had three equations per regression analysis: the associations of cfPWV with each of LVMI^2.7^, RWT, LVDF, and LVFP (Equation 1); the associations of cumulative total body fat mass, lean mass, systolic BP, heart rate or insulin resistance (Equation 2); and the associations of cfPWV with cumulative each of LVMI^2.7^, RWT, LVDF, and LVFP (Equation 3, total effect), and Equation 3′ (direct effect) accounted for the mediating role of total body fat mass, lean mass, systolic BP, heart rate or insulin resistance on the longitudinal associations of cfPWV with LVMI^2.7^, RWT, LVDF, and LVFP. The proportion of mediating or suppressing roles was estimated as the ratio of the difference between Equation 3 and Equation 3’ or the multiplication of Equations 1 and 2 divided by Equation 3 and expressed in percentage. A mediating or indirect role is confirmed when there are statistically significant associations between (a) the predictor and mediator, (b) the predictor and outcome, (c) the mediator and outcome, and when (d) the longitudinal associations between the predictor and outcome variable was attenuated upon inclusion of the mediator [42,44]. However, when the magnitude of the longitudinal association between the predictor and outcome is increased upon inclusion of a third variable, a suppression is confirmed [44]. Path analyses were conducted with 1000 bootstrapped samples.

Differences and associations with a 2-sided *p*-value <0.05 were considered statistically significant with conclusions based on effect estimates and their confidence intervals or standard errors. Analyses involving 20% of a sample of 10,000 ALSPAC children at 0.8 statistical power, 0.05 alpha, and 2-sided *p*-value would show a minimum detectable effect size of 0.062 standard deviations if they had relevant exposure for a normally distributed quantitative variable [45]. Missing data were handled with multiple imputations as it has >98% efficiency in simulating real data [8,9,31,46] Multiple comparisons were adjusted for using sequential Sidak correction. All statistical analyses were performed using SPSS statistics software, Version 27.0 (IBM Corp, Armonk, NY, USA), and structural equation modeling was conducted using IBM AMOS version 27.0.

## 3. Results

### 3.1. Cohort study characteristics

We studied 1856 participants who had complete cfPWV, cIMT, LVMI^2.7^, and RWT measurements at the age 17 years (Supplemental Fig. 1). Males had higher cfPWV, cIMT, LVMI^2.7^, RWT, and LVDF than females at both 17 and 24 years, however, females had higher LVFP than males at both observation periods. The prevalence of LVH and hiLVFP doubled in both males and females during the 7-year observation period. The prevalence of hiRWT decreased by half in both sexes while that of LVDD decreased significantly in males but doubled in females. Other participants’ characteristics are shown in Table 1 and Supplemental Table 1.

### 3.2. Determinants of cardiac structure and function progression

The positive determinants of cumulatively higher LVMI^2.7^ progression were cumulatively increased total fat mass, lean mass, systolic BP, and moderate to vigorous physical activity (Table 2). The cumulative determinant of RWT progression was increased smoking, LVDF progression was lower heart rate, and LVFP progression were female sex, reduced moderate to vigorous physical activity, and higher socioeconomic status (Table 2).

### 3.3. Temporal path associations between vascular and cardiac indices

**Fig. 1.**
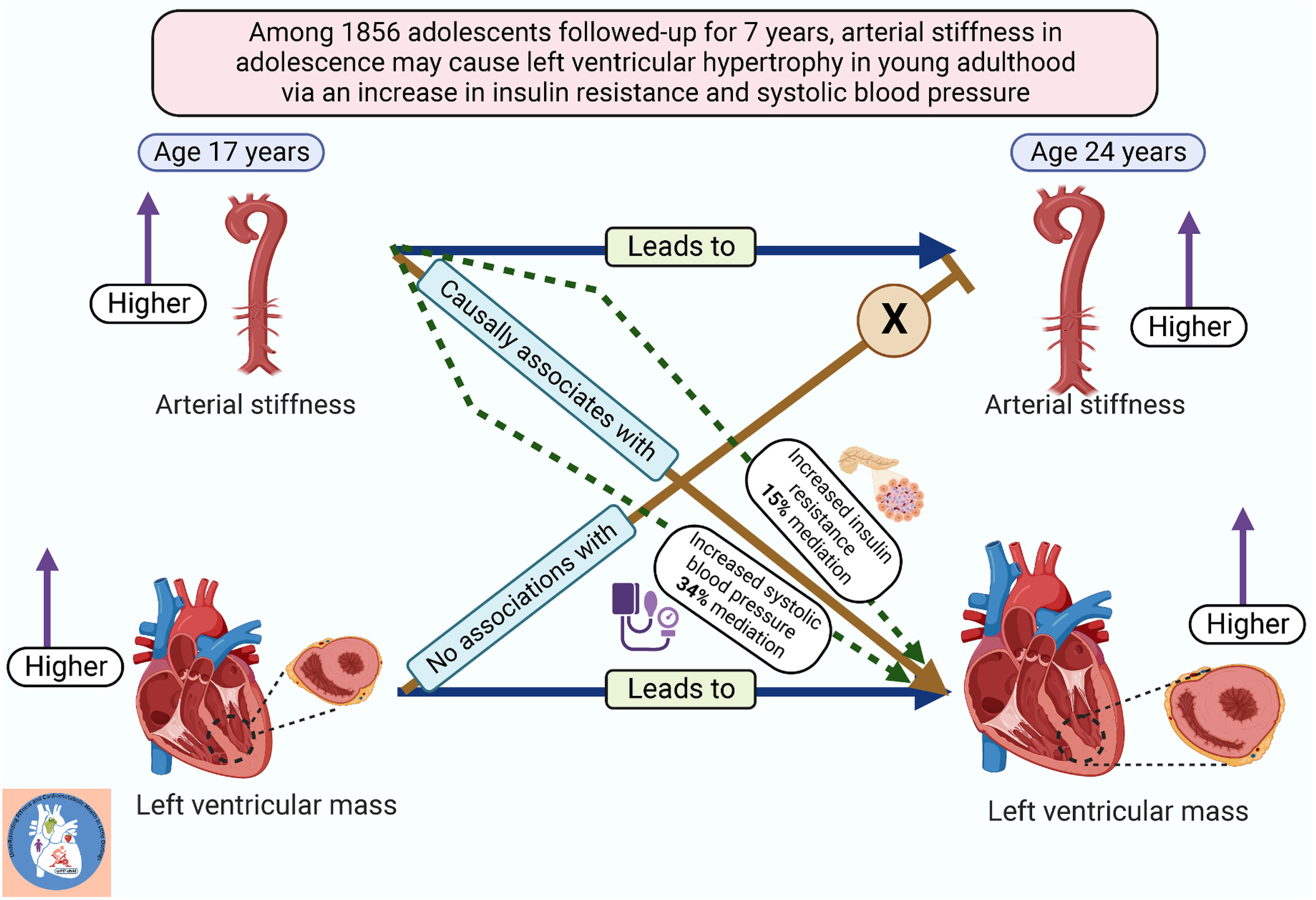
Temporal causal longitudinal relationships between arterial stiffness and left ventricular mass with the mediating role of insulin resistance and systolic blood pressure. Auto-regressive cross-lagged temporal causal associations of arterial stiffness with left ventricular mass index among 1856 adolescents aged 17 years and followed up until age 24 years from the Avon Longitudinal Study of Parents and Children birth cohort, England, UK.

Higher cfPWV, cIMT, LVMI^2.7^, RWT, LVDF, and LVFP at 17 years were directly associated with their individual variables at 24 years. Higher cfPWV at baseline was directly associated with higher LVMI^2.7^, RWT, and LVFP and negatively associated with LVDF at follow-up. However, higher cardiac indices at baseline were not associated with higher cfPWV at follow-up (Table 3 and Fig. 1). There were no statistically significant associations between higher cIMT at baseline and higher cardiac indices at follow-up and *vice versa* (Table 3). However, higher cIMT at baseline had a borderline significant association with higher LVDF at follow-up (*p* = 0.05).

### 3.4. Longitudinal predictive associations between vascular and cardiac damage

After adjustment for cardiometabolic and lifestyle risk factors, cumulative high cfPWV progression was associated with an increased risk of worsening LVH progression, both in the total cohort and among males, and an increased risk of worsening hiLVFP in the total cohort and among females (Table 4 and Supplemental Table 2). High cfPWV progression was not associated with the risk of worsening hiRWT and LVDD, in the total cohort but was associated with decreased risk of hiRWT in both males and females. In normotensive individuals, cumulative high cfPWV progression was associated with an increased risk of worsening LVH and a decreased risk of worsening hiLVFP. Among participants with elevated systolic BP/hypertension, cumulative high cfPWV progression was associated with decreased risk of worsening LVH and hiRWT (Supplemental Table 3). In normal-weight individuals, cumulative high cfPWV progression was associated with an increased risk of worsening LVDD and hiLVFP, while among overweight/obese individuals, cumulative high cfPWV progression was associated with an increased risk of worsening LVH (Supplemental Table 4).

Cumulative moderate and high cIMT progression was associated with an increased risk of worsening LVH in the total cohort and overweight/obese participants (Table 4 and Supplemental Table 4). In both males and females and normal-weight participants, cumulative high cIMT progression was associated with decreased risk of worsening hiLVFP (Supplemental Tables 4 and 5). In the total cohort and among participants with elevated systolic BP/hypertension, high cIMT progression was associated with an increased risk of worsening hiLVFP (Table 4 and Supplemental Table 3). Cumulative high cIMT progression was associated with a 66% increased risk of worsening hiRWT progression among participants with elevated systolic BP/hypertension (Supplemental Table 3). Analyses with complete arterial and cardiac variables at both baseline and follow-up were largely in consonance with the imputed findings (Supplemental Table 5).

### 3.5. Mediation path analyses of arterial stiffness with cardiac structure and function

Cumulative total body fat mass from ages 17–24 years partly suppressed the associations of cumulatively increased cfPWV with increased LVMI^2.7^ (18.4% suppression effect), RWT (5% suppression effect) and decreased LVDF (25.8% suppression effect) but not LVFP (Table 5). Cumulative lean mass and insulin resistance from ages 17 to 24 years partly mediated the associations of cumulatively increased cfPWV with increased LVMI^2.7^ and RWT (Table 5). Cumulative systolic BP from ages 17 to 24 years partly mediated the associations of cumulatively increased cfPWV with increased LVMI^2.7^ (34.3% mediation effect) (Table 5 and Fig. 1). Cumulative heart rate had neither mediation nor suppression effects on the associations of vascular and cardiac indices (Table 5).

## 4. Discussion

Given the infeasibility of decades-long randomized clinical trials in a paediatric population aimed at diseases development, carefully collected cohort study data of changes across time in a predictor and in an outcome offers evidence of a possible causal role, when bolstered with biological plausibility. The presented data is the first to demonstrate a temporal longitudinal relationship of arterial changes, assessed as stiffness with cfPWV and thickness with cIMT *versus* repeated measures of cardiac structure and function in a substantial population of adolescents and young adults, provides evidence that arterial stiffness may be a causal risk factor for the development of cardiac damage independent of BP and obesity, identifies the distinguishing role of arterial stiffness in concentric and eccentric ventricular remodelling, and the sex-based distinction in ventriculo-arterial relationship, in addition to the mediating role of adiposity and BP. First, we observed that longitudinal determinants of LVMI^2.7^ progression in youth were lean mass, fat mass, systolic BP, and moderate to vigorous physical activity. Next, we reported, using cross-lagged temporal causal models, that arterial stiffness, but not carotid thickness, may temporally precedes increased alterations in cardiac structure and function. We also observed that cumulatively high cfPWV and cIMT over 7 years were associated with an increased risk of worsening cardiac structural and functional damage in youth. Lastly, we observed that increased systolic BP mediated one-third of the likely causal relationship between arterial stiffness and increased cardiac mass.

Several cross-sectional, prospective, and clinical adult studies have established the role of arterial stiffness in ventriculo-arterial coupling, ventricular remodelling, incident LVH, heart failure, LVDD, and other cardiovascular events with mechanistic studies confirming these reports [3,4,13,19,21,47,48]. There is grossly limited knowledge on the longitudinal relationships of arterial stiffness with cardiac structure and function in the youth, with a few cross-sectional studies conducted in a relatively small to moderate-sized population [22,23,49]. It has been consistently reported that in young adults, cfPWV is low, and reflected waves usually return to the aortic root during diastole but with increasing age around middle-aged adulthood, cfPWV increases and reflected waves arrive in mid-late systole leading to altered ventricular structure and function and consequent cardiac events [3,4,13]. Also, in 50-year-old adults, baseline cfPWV was associated with progressing LV concentric remodelling [50]. However, we observed that a high tertile category of cfPWV in a general population of healthy growing adolescents was already associated with adverse cardiac remodelling and functional alterations 7 years later, just as baseline cfPWV was associated with higher LVMI^2.7^, RWT, LVFP, and lower LVDF. Childhood and adolescent obesity and BP have been reported as early life determinants of single-measured middle-aged LVH [5–7]. The current longitudinal study extends this evidence by revealing that cumulative fat mass and systolic BP were strong determinants LVMI^2.7^ progression over 7 years and this could be clinically relevant since early life systolic BP and obesity have been associated with premature death [2,46,51].

Regarding sex-specific differences, cumulative high cfPWV was associated with worsening LVH in males but not in females, whereas cumulative moderate to high cfPWV was associated with worsening LVDD in females only. The susceptibility of adult women to microvascular dysfunction and heart failure with preserved ejection fraction has been reported [52], with men being predisposed to macrovascular diseases or heart failure with reduced ejection fraction. The female heart is smaller in size compared to males [37] and has lesser stroke volume but higher heart rate, which ensures adequate cardiac output. The single LVH cutpoint of 51 g/m^2.7^ that has been associated with clinical events [41] might have identified fewer females with LVH at both time points resulting in reduced statistical power for detecting a difference [37]. Cumulative high cfPWV progression was directly associated with an increased risk of worsening structural cardiac damage among overweight/obese individuals but associated with an increased risk of worsening functional cardiac damage in normal-weight individuals. Among normotensive individuals, cumulative high cfPWV progression was directly associated with an increased risk of worsening cardiac structural damage. However, among participants with elevated systolic BP/hypertension, the relationship between high cfPWV progression and worsening cardiac structural damage was inverse, plausibly due to vascular physiologic adaptation where high arterial stiffness suppress cardiac structural damage in the presence of elevated systolic BP [43].

Our findings suggest that the sex distinction in cardiac anatomy and physiology already contributes to cardiovascular disease development beginning in adolescence and that irrespective of obesity status, arterial stiffness progression contributes to either structural or functional progressive cardiac damage. It is pertinent to note that while systolic BP partly mediated the causal path between arterial stiffness and increased ventricular mass, total fat mass suppressed the relationship. This suggests that adiposity may not be directly implicated in the causal path of the deleterious effect of arterial stiffness on cardiac structure and function. However, since insulin resistance partly mediated the associations between arterial stiffness and altered cardiac structure and function, fat mass may indirectly exerted its influence through this pathway, due to the strong predictive relationships between adiposity and insulin resistance [53]. Together, systolic BP and insulin resistance had an almost 50% mediation effect on the relationship between arterial stiffness and increased ventricular mass. It is known that arterial stiffness may causally raise BP via higher insulin resistance in adolescents and low-grade inflammation may causally increase arterial stiffness [10, 14,54]. Thus, mechanistic delineation of coupling in ventriculo-arterial remodelling and consequent physiologic dysfunction is warranted [48].

A meta-analysis of randomized controlled trials in adults concluded that cIMT progression is a surrogate marker for cardiovascular disease risk and that the extent of intervention effects on cIMT progression predicted the degree of cardiovascular disease risk reduction [55]. Several paediatric studies have utilized cIMT as a subclinical marker of atherosclerotic cardiovascular diseases in middle-aged adulthood, but longitudinal data in adolescence linking cIMT to early organ damage in young adulthood are non-existent [16,18,28]. A Chinese study among middle-aged adults (36–45-year-olds) showed that higher cIMT was cross-sectionally associated with the risk of LVH but was not statistically significant in the 4-year longitudinal analysis, likely due to their diagnosis of LVH with an electrocardiogram [56]. We observed that cumulative high tertiles of cIMT progression from adolescence were associated with the risk of worsening LVH and hiLVFP progression in the total cohort, but this was not statistically significant in separate analyses of either males or females. Importantly, in both overweight/obese and elevated systolic BP/hypertensive participants, high tertile of cIMT progression was associated with worsening cardiac structural damage. We did not observe a temporal time sequence in the relationship between repeated measures of cIMT and cardiac structure and function, warranting further studies with longer follow-ups.

Taken together, from the perspective of primordial and primary prevention, the arterial path to cardiovascular diseases and event risk in adulthood appears to be informative. It is of public health concern that cumulative high cfPWV progression was associated with LVH progression independent of several established cardiometabolic and lifestyle factors, signalling attention to drivers of arterial stiffness in early life [10]. The novel temporal causal role of arterial stiffness in cardiovascular disease development in normotensive and normal-weight youths calls for clinical and public health intervention to potentially attenuate and reverse the progression of arterial stiffness [8–10]. The presence of overweight/obesity and elevated systolic BP/hypertension as the basis of the relation between cIMT progression and worsening LVH emphasizes the need to continue targeting these major risk factors from early life. Hence, the assessment of arterial stiffness in the general population should begin in adolescence, and a reduction in risk-factor levels including arterial stiffness before early adulthood may attenuate the incidence of premature cardiovascular events in mid-life [2,10,13]. Of note, reducing elevated or high BP in youth without concurrently attenuating or treating the causal role of arterial stiffness may lead to inefficient reversal or delay in cardiac damage, since arterial stiffness may causally predict elevated BP in youth [8].

Using an extensively phenotyped large birth cohort (ALSPAC), we applied advanced statistical tools to improve our understanding of a potential causal path in the ventriculo-arterial relationship. The ALSPAC data provides ample normative data relevant to general clinical paediatric practice. We controlled for objectively and repeatedly measured confounders such as physical activity, sedentary time, fat mass, lean mass, and several lifestyle factors like smoking, family history, and socioeconomic status. Nonetheless, we may not exclude the existence of residual biases from unmeasured confounders, unusual reverse causation, or collider effects. This study fills the knowledge gap on the physiological and pathological cardiovascular alterations occurring during adolescence and young adulthood before clinical events occur in middle age or later in life [2,16,18]. The use of adult thresholds may not be calibrated for earlier life course time points [37], but we elected to use these thresholds based on clinical guidelines. Our participants were mostly Caucasians with minimal geographic variations; thus, our findings may not be generalizable to other ethnicities.

### 4.1. Conclusion

In a 7-year prospective assessment of the temporal causal relationships of arterial stiffness and carotid thickness progression with structural and functional cardiac organ damage, we observed that arterial stiffness progression temporally preceded and may be causally associated with increased risk of worsening structural and functional cardiac damage in both males and females, irrespective of obesity and hypertensive state. cIMT progression was associated with an increased risk of worsening LVH, particularly among overweight/obese and elevated systolic BP/hypertensive participants. The cumulative longitudinal determinants of LVMI^2.7^ progression in youth were increasing fat mass, lean mass, and systolic BP. The prevalence of LVH doubled during the observation period. Arterial stiffness may potentially cause cardiac structural damage via an increase in systolic BP and insulin resistance. Primordial and primary prevention of arterial stiffness in youth and mechanistic delineation of coupling in ventriculo-arterial remodelling and consequent physiologic dysfunction is warranted. Future intervention studies investigating the effect of simultaneous reduction of arterial stiffness and elevated BP on the risk of target cardiac structural and functional damage in youth are needed.

## Supplementary Material

supplemental material

Appendix A. Supplementary data

Supplementary data to this article can be found online at https://doi.org/10.1016/j.atherosclerosis.2023.117197.

## Figures and Tables

**Table 1:** Descriptive characteristics of cohort participants.

	17 years					24 years				
Variables	Male		Female		*p*-value	Male		Female		*p-*value

	N	Mean (SD)	N	Mean (SD)		N	Mean (SD)	N	Mean (SD)	

**Anthropometry**										
Age (years)	845	17.69 (0.32)	1011	17.69 (0.32)	0.990	528	24.69 (0.63)	759	24.61 (0.63)	0.029
Height (m)	845	1.79 (0.07)	1011	1.65 (0.06)	<0.0001	526	1.80 (0.07)	747	1.66 (0.06)	<0.0001
*Weight (kg)	845	69 (14.10)	1011	61 (14.10)	<0.0001	525	77.20 (17.65)	748	64.75 (18.17)	<0.0001
Ethnicity- White (n,%)	782	746 (95.4)	909	875 (96.3)	0.393	NA				
**Body composition**										
*Total fat mass (kg)	832	10.47 (9.98)	999	19.74 (10.81)	<0.0001	509	17.72 (10.12)	723	22.06 (12.85)	<0.0001
*Lean mass (kg)	832	54.51 (7.76)	999	37.87 (5.40)	<0.0001	509	55.82 (10.04)	723	40.93 (7.31)	<0.0001
*Body mass index (kg/m^2^)	845	21.48 (4.20)	1011	22.29 (4.70)	<0.0001	525	23.82 (4.64)	747	23.65 (6.39)	0.620
**Fasting plasma metabolic indices**										
High-density lipoprotein (mmol/L)	647	1.20 (0.26)	646	1.35 (0.32)	<0.0001	474	1.43 (0.38)	602	1.69 (0.44)	<0.0001
Low-density lipoprotein (mmol/L)	647	2.00 (0.56)	646	2.18 (0.62)	<0.0001	473	2.49 (0.79)	602	2.41 (0.74)	0.087
*Triglyceride (mmol/L)	647	0.75 (0.34)	646	0.75 (0.37)	0.458	473	0.88 (0.52)	602	0.83 (0.47)	0.001
Glucose (mmol/L)	647	5.18 (0.54)	646	4.92 (0.37)	<0.0001	474	5.54 (0.63)	602	5.31 (0.62)	<0.0001
*Insulin (mU/L)	637	6.08 (3.95)	638	7.66 (4.52)	<0.0001	474	7.15 (4.99)	602	8.0 (5.42)	0.001
*High sensitivity C-reactive protein (mg/L)	647	0.44 (0.73)	646	0.65 (1.28)	<0.0001	434	0.63 (1.13)	572	1.02 (2.03)	<0.0001
**Vascular measures**										
Heart rate (beat/mins)	845	62 (9)	1009	67 (10)	<0.0001	526	64 (10)	757	69 (10)	<0.0001
Systolic blood pressure (mm Hg)	845	120 (9)	1009	110 (8)	<0.0001	526	122 (11)	757	111 (9)	<0.0001
Diastolic blood pressure (mm Hg)	845	63 (6)	1009	65 (6)	<0.0001	526	67 (8)	757	66 (8)	0.382
*Carotid-femoral PWV (m/s)	845	5.99 (0.82)	1011	5.53 (0.75)	<0.0001	388	6.53 (1.27)	588	5.97 (1.05)	<0.0001
*Carotid IMT (mm)	845	0.48 (0.06)	1011	0.47 (0.06)	<0.0001	312	0.52 (0.10)	491	0.52 (0.08)	0.059
**Cardiac measures**										
Left ventricular mass indexed for height (g/m^2.7^)	845	37.61 (7.98)	1011	34.86 (7.08)	<0.0001	330	40.67 (8.61)	509	36.96 (7.91)	<0.0001
Left ventricular hypertrophy (n,%)	845	47 (5.6)	1011	20 (2.0)	<0.0001	330	39 (11.8)	509	21 (4.1)	<0.0001
Relative wall thickness (cm)	845	0.38 (0.06)	1011	0.38 (0.06)	0.121	331	0.37 (0.05)	509	0.36 (0.05)	0.008
Increased relative wall thickness (n,%)	845	102 (12.1)	1011	107 (10.6)	0.338	331	27 (8.2)	509	27 (5.3)	0.113
Left ventricular diastolic function (E/A)	820	1.95 (0.38)	971	1.92 (0.41)	0.055	343	2.01 (0.54)	527	2.01 (0.58)	0.965
Left ventricular diastolic dysfunction (n,%)	820	75 (9.1)	971	124 (12.8)	0.016	343	58 (16.9)	527	84 (15.9)	0.708
Left ventricular filling pressure (LVFP) (E/e’)	816	4.86 (1.08)	965	4.88 (0.97)	0.808	319	4.84 (0.98)	509	5.19 (1.02)	<0.0001
Increased LVFP (n,%)	816	13 (1.6)	965	6 (0.6)	0.062	319	1 (0.3)	502	5 (1.0)	0.415
**Lifestyle factors**										
Smoked cigarette ever (n,%)	762	314 (41.2)	907	449 (49.5)	0.001	522	320 (61.3)	755	452 (59.9)	0.641
Sedentary time (mins/day)	397	466 (88)	476	486 (79)	<0.0001	143	529 (82)	241	526 (84)	0.726
LPA (mins/day)	397	290 (70)	476	273 (60)	<0.0001	143	148 (63)	241	156 (58)	0.252
MVPA (mins/day)	397	56 (30)	476	40 (23)	<0.0001	143	52 (30)	241	48 (28)	0.235
Family history of H-D-C-V (n,%)	845	259 (30.7)	1010	326 (32.3)	0.482	NA				
Maternal social economic status (n,%)	400		416		0.971	NA				
Professional		33 (8.3)		25 (6.0)						
Managerial and technical		155 (38.8)		156 (37.5)						
Skilled non-manual		137 (34.3)		148 (35.6)						
Skilled manual		4 (1.0)		7 (1.7)						
Partly skilled		61 (15.3)		63 (15.1)						
Unskilled		10 (2.5)		17 (4.1)						

The values are means (standard deviations) and *median (interquartile range) except for lifestyle factors, cardiac structure and function risk categories in percentage. Differences between sexes were tested using Student’s t-test for normally distributed continuous variables, Mann–Whitney *U* test for skewed continuous variables, and Chi-square test for dichotomous variable. A 2-sided *p*-value <0.05 is considered statistically significant. Participants with LVMI^2.7^ ≥51 g/m^2.7^, relative wall thickness ≥0.44, LVDD ≥1.5 and LVFP ≥8 were categorized as at risk of altered cardiac structure and function. LPA, light physical activity; MVPA, moderate to vigorous physical activity; NA, not available/applicable; PWV, pulse wave velocity; *p*-value for sex differences.

**Table 2:** Age 17 years through 24 years cumulative determinants of cardiac structure and function progression from adolescence (17 years) through young adulthood (24 years).

N = 1856	Left ventricular mass index^2.7^		Relative wall thickness		Left ventricular diastolic function (E/A)		Left ventricular filling pressure (E/e’)	
Predictor	Effect estimate (95% CI)	*p*-value	Effect estimate (95% CI)	*p*-value	Effect estimate (95% CI	*p*-value	Effect estimate (95% CI	*p*-value

Intercept	44.119 (−24.85–113.09)	0.209	−0.021 (−0.590–0.548)	0.942	5.074 (0.169–9.980)	**0.043**	0.699 (−9.543–10.940)	0.893
Sex	0.453 (−3.005–3.911)	0.797	0.016 (−0.012–0.044)	0.271	0.011 (−0.236–0.259)	0.927	0.667 (0.158–1.177)	**0.011**
Age	−4.232 (−7.145–−1.319)	**0.005**	−0.001 (−0.025–0.023)	0.949	−0.098 (−0.298–0.103)	0.338	−0.034 (−0.461–0.393)	0.876
High-density lipoprotein cholesterol	−0.758 (−3.088–1.571)	0.522	−0.011 (−0.030–0.008)	0.254	0.049 (−0.121–0.220)	0.570	0.082 (−0.265–0.428)	0.644
Low-density lipoprotein cholesterol	−0.185 (−1.630–1.261)	0.802	0.003 (−0.009–0.015)	0.582	−0.097 (−0.203–0.010)	0.074	0.021 (−0.196–0.238)	0.849
Triglyceride	1.492 (−4.434–7.418)	0.620	−0.016 (−0.064–0.033)	0.524	−0.094 (−0.521–0.333)	0.666	0.731 (−0.153–1.615)	0.105
High-sensitivity C-reactive protein	1.333 (−0.333–2.999)	0.116	0.002 (−0.012–0.016)	0.768	0.022 (−0.104–0.147)	0.734	−0.097 (−0.351–0.158)	0.455
Insulin	−2.477 (−6.762–1.808)	0.256	0.007 (−0.028–0.042)	0.702	−0.077 (−0.400–0.247)	0.640	−0.442 (−1.091–0.208)	0.182
Glucose	−5.996 (−2.749–1.549)	0.583	−0.011 (−0.028–0.006)	0.518	−0.028 (−0.196–0.141)	0.747	0.187 (−0.149–0.524)	0.273
Total fat mass	12.699 (8.055–17.344)	**<0.0001**	0.026 (−0.012–0.064)	0.186	−0.026 (−0.375–0.323)	0.884	−0.688 (−1.398–0.021)	0.057
Lean mass	26.976 (8.113–45.840)	**0.005**	0.069 (−0.086–0.224)	0.380	0.108 (−1.253–1.468)	0.876	2.006 (−0.812–4.823)	0.162
Systolic blood pressure	0.144 (0.023–0.265)	**0.020**	0.001 (−0.001–0.002)	0.265	0.005 (−0.006–0.013)	0.435	0.003 (−0.016–0.021)	0.781
Diastolic blood pressure	0.042 (−0.125–0.210)	0.620	0.001 (−0.001–0.002)	0.333	0.0001 (−0.013–0.013)	0.997	0.016 (−0.010–0.041)	0.229
Heart rate	−0.138 (−0.223–−0.052)	**0.002**	0.0002 (−0.001–0.001)	0.518	−0.017 (−0.018–−0.005)	**<0.0001**	−0.011 (−0.024–0.002)	0.098
Sedentary time	0.001 (−0.010–0.011)	0.935	0.0001 (−0.001–0.0001)	0.156	−0.001 (−0.001–0.0003)	0.230	−0.001 (−0.002–0.001)	0.336
Light physical activity	−0.010 (−0.021–0.002)	0.091	0.0001 (−0.0001–0.0001)	0.872	−0.001 (−0.002–0.0003)	0.164	0.0001 (−0.002–0.002)	0.901
Moderate to vigorous physical activity	0.036 (0.007–0.066)	**0.016**	−0.0001 (−0.0003–0.0002)	0.562	−0.001 (−0.003–0.001)	0.348	−0.007 (−0.012–−0.003)	**0.002**
Socio-economic status	−0.552 (−1.212–0.109)	0.101	−0.001 (−0.007–0.004)	0.706	−0.037 (−0.084–0.010)	0.124	0.111 (0.013–0.209)	**0.026**
Family history of cardiometabolic disease	−0.442 (−2.238–1.355)	0.629	−0.003 (−0.018–0.011)	0.650	0.020 (−0.108–0.147)	0.759	0.044 (−0.224–0.312)	0.746
Smoking status	0.636 (−0.648–1.921)	0.329	0.012 (0.001–0.022)	**0.029**	−0.071 (−0.174–0.031)	0.170	0.190 (−0.488–0.867)	0.581
Ethnicity	−0.092 (−4.669–4.486)	0.969	0.027 (−0.010–0.065)	0.155	−0.268 (−0.591–0.055)	0.104	−0.158 (−0.356–0.040)	0.116

Analyses were conducted using linear mixed-effect models. All covariates were forced into the same model and statistically significant covariates were determined by a 2-sided *p*-value <0.05.

**Table 3:** Auto-regressive cross-lagged temporal causal path analyses of carotid-femoral pulse wave velocity and carotid intima-media thickness with cardiac damage risk at 17 and 24 years of age.

1856 participants	Carotid-femoral pulse wave velocity					Carotid intima-media thickness					

Auto-regressive		B	β	SE	*p*-value			B	β	SE	*p*-value

LVMI^2.7^ T1	⇨ LVMI^2.7^ T2	0.566	0.506	0.035	**<0.0001**	LVMI^2.7^ T1	⇨ LVMI^2.7^T2	0.562	0.502	0.035	**<0.0001**
RWT T1	⇨ RWT T2	0.227	0.249	0.031	**<0.0001**	RWT T1	⇨ RWT T2	0.235	0.259	0.031	**<0.0001**
LVDF T1	⇨ LVDF T2	0.408	0.284	0.048	**<0.0001**	LVDF T1	⇨ LVDF T2	0.412	0.287	0.048	**<0.0001**
LVFP T1	⇨ LVFP T2	0.327	0.327	0.033	**<0.0001**	LVFP T1	⇨ LVFP T2	0.326	0.326	0.033	**<0.0001**
cfPWV T1	⇨ cfPWV T2	0.484	0.337	0.046	**<0.0001**	cIMT T1	⇨ cIMT T2	0.384	0.294	0.044	**<0.0001**
**Cross-lagged**											
LVMI^2.7^ T1	⇨ cfPWV T2	0.0001	0.005	0.0001	0.870	LVMI^2.7^ T1	⇨ cIMT T2	0.0001	0.022	0.0001	0.553
cfPWV T1	⇨ LVMI^2.7^ T2	10.865	0.064	5.143	**0.035**	cIMT T1	⇨ LVMI^2.7^ T2	−3.176	−0.015	5.743	0.580
RWT T1	⇨ cfPWV T2	−0.030	−0.024	0.038	0.429	RWT T1	⇨ cIMT T2	0.052	0.055	0.032	0.109
cfPWV T1	⇨ RWT T2	0.094	0.091	0.034	**0.006**	cIMT T1	⇨ RWT T2	0.037	0.030	0.886	0.376
LVDF T1	⇨ cfPWV T2	0.0001	0.003	0.006	0.934	LVDF T1	⇨ CIMT T2	−0.002	−0.015	0.005	0.662
cfPWV T1	⇨ LVDF T2	−0.716	−0.063	0.362	**0.048**	cIMT T1	⇨ LVDF T2	0.865	0.063	0.442	0.050
LVFP T1	⇨ cfPWV T2	0.001	0.013	0.002	0.665	LVFP T1	⇨ cIMT T2	−0.001	−0.025	0.002	0.469
cfPWV T1	⇨ LVFP T2	2.225	0.109	0.707	**0.002**	cIMT T1	⇨ LVFP T2	0.353	0.014	0.808	0.663

Time T1, 17.7 years of age; Time T2, 24.5 years; B, unstandardized regression; *β*, standardized regression, SE, standard error; cfPWV, carotid-femoral pulse wave velocity; cIMT, carotid intima-media thickness; LVDF, left ventricular diastolic function (E/A); LVFP, left ventricular filling pressure (E/e’); LVMI^2.7^, left ventricular mass indexed for height^2.7^; PWV, pulse wave velocity; RWT, relative wall thickness. Autoregressive cross-lagged temporal path analyses were adjusted for statistically significant determinants of cardiac structure and function progression described in Table 2. Skewed variables were logarithmically transformed before analyses. A 2-sided *p*-value <0.05 is considered statistically significant.

**Table 4:** Effect of arterial stiffness and carotid intima-media thickness progression from ages 17 through 24 years on the risk of worsening left ventricular hypertrophy and diastolic dysfunction from ages 17 through 24 years.

N = 1856	Worsening LVH		Increasing RWT		Worsening LVDD		Increasing LVFP	
	Odds ratio (95% CI)	*p*-value	Odds ratio (95% CI)	*p*-value	Odds ratio (95% CI)	*p*-value	Odds ratio (95% CI)	*p*-value

**Carotid-femoral pulse wave velocity progression**								
Low category	Reference	–	Reference	–	Reference	–	Reference	–
Moderate category	0.94 (0.84–1.05)	0.258	0.97 (0.66–1.43)	0.873	1.10 (0.88–1.37)	0.416	1.01 (0.93–1.09)	0.841
High category	1.23 (1.13–1.35)	**<0.001**	0.90 (0.75–1.07)	0.218	1.13 (0.97–1.31)	0.126	1.14 (1.13–1.14)	**<0.0001**
**Carotid intima-media thickness progression**								
Low category	Reference	–	Reference	–	Reference	–	Reference	–
Moderate category	1.36 (1.36–1.37)	**<0.0001**	0.88 (0.81–0.96)	**0.005**	1.04 (0.96–1.14)	0.352	1.08 (1.03–1.13)	**0.002**
High category	1.27 (1.26–1.27)	**<0.0001**	1.01 (0.75–1.36)	0.959	1.13 (0.87–1.47)	0.348	1.17 (1.07–1.28)	**<0.001**

Multivariable analyses were adjusted for sex, time in years between ages 17.7 and 24.5 years, age at baseline, and other time-varying covariates measured at both baseline and follow-up such as low-density lipoprotein cholesterol, insulin, triglyceride, high-sensitivity C reactive protein, high-density lipoprotein cholesterol, heart rate, glucose, systolic blood pressure, fat mass, lean mass, smoking status, family history of hypertension/diabetes/high cholesterol/vascular disease, social-economic status, and moderate to vigorous physical activity, light physical activity and sedentary time at 15 and 24 years.

Skewed predictors and covariates were logarithmically transformed before analyses. Odds ratio are follow-up time X predictor interaction effect computed using generalized logit mixed-effect model for repeated measures; CI, confidence interval; LVDD, left ventricular diastolic dysfunction; LVFP, left ventricular filling pressure; LVH, left ventricular hypertrophy; LVMI^2.7^, left ventricular mass indexed for height^2.7^. A 2-sided *p*-value <0.05 is considered statistically significant. Multiple testing was corrected with Sidak correction. Predictors were categorized in tertiles: tertile 1 is low, tertile 2 as moderate, and tertile 3 as high categories. Participants with LVMI^2.7^ ≥51 g/m^2.7^, relative wall thickness ≥0.44, LVD function <1.5, and LVFP ≥8 were categorized as having LVH, increased RWT, LVDD and increased LVFP respectively. Multiple imputations were used to account for missing variables.

**Table 5:** Mediating or suppressing role of body composition, blood pressure, and insulin resistance on the longitudinal associations of arterial stiffness with cardiac structure and function.

Predictor: Carotid-femoral pulse wave velocity							

(N = 1856)	Total effect		Direct effect		Indirect effect		Mediation (%)
Mediators	β (95% CI)	*p*-value	β (95% CI)	*p*-value	β (95% CI)	*p*-value	

**Left ventricular mass index**							
Total fat mass	0.272 (0.107–0.472)	**0.001**	0.322 (0.128–0.551)	**0.001**	−0.050 (−0.093–−0.018)	**0.000**	**18.4** suppression
Lean mass	0.290 (0.049–0.534)	**0.001**	0.250 (0.027–0.463)	**0.002**	0.041 (0.017–0.076)	**0.001**	**14.1** mediation
Systolic BP	0.251 (0.087–0.428)	**0.001**	0.165 (0.050–0.283)	**0.001**	0.086 (0.031–0.155)	**0.001**	**34.3** mediation
Heart rate	0.292 (0.122–0.492)	**0.001**	0.300 (0.123–0.505)	**0.001**	−0.008 (−0.028–0.001)	0.086	2.7
Insulin resistance	0.291 (0.119–0.488)	**0.001**	0.247 (0.099–0.417)	**0.001**	0.044 (0.016–0.083)	**0.000**	**15.1** mediation
**Relative wall thickness**							
Total fat mass	0.060 (0.019–0.115)	**0.001**	0.063 (0.024–0.118)	**0.001**	−0.003 (−0.007–0.000)	**0.043**	**5.0** suppression
Lean mass	0.048 (0.005–0.108)	**0.029**	0.044 (0.000–0.106)	0.053	0.004 (0.001–0.007)	**0.017**	**8.3** mediation
Systolic BP	0.061 (−0.002–0.119)	0.059	0.009 (−0.023–0.039)	0.458	0.052 (0.017–0.104)	0.001	85.3
Heart rate	0.066 (0.023–0.108)	**0.001**	0.058 (0.021–0.093	**0.002**	0.008 (−0.001–0.024)	0.116	12.1
Insulin resistance	0.084 (0.019–0.153)	**0.001**	0.064 (0.010–0.115)	**0.007**	0.020 (0.007–0.044)	**0.000**	**23.8** mediation
**Left ventricular diastolic function**							
Total fat mass	−0.031 (−0.071–−0.054)	**0.003**	−0.039 (−0.078–0.003)	0.070	0.008 (0.005–0.013)	**0.001**	**25.8** suppression
Lean mass	−0.044 (−0.079–0.002)	0.061	−0.047 (−0.084–0.001)	0.057	0.003 (0.000–0.006)	0.026	6.8
Systolic BP	−0.020 (−0.056–0.025)	0.356	−0.012 (−0.050–0.035)	0.614	−0.008 (−0.014–−0.003)	0.002	40.0
Heart rate	−0.026 (−0.067–0.022)	0.346	−0.024 (−0.065–0.021)	0.334	−0.003 (−0.012–0.008)	0.665	11.5
Insulin resistance	−0.028 (−0.064–0.012)	0.165	−0.020 (−0.055–0.022)	0.357	−0.008 (−0.013–−0.004)	0.001	28.6
**Left ventricular filling pressure**							
Total fat mass	0.021 (−0.016–0.058)	0.229	0.020 (−0.018–0.057)	0.273	0.001 (−0.002–0.005)	0.597	4.8
Lean mass	0.037 (−0.001–0.073)	0.054	0.041 (0.002–0.078)	**0.041**	−0.004 (−0.008–−0.002)	0.001	10.8
Systolic BP	0.014 (−0.026–0.052)	0.474	0.009 (−0.032–0.051)	0.622	0.005 (−0.001–0.010)	0.102	35.7
Heart rate	0.020 (−0.016–0.057)	0.257	0.020 (−0.016–0.057)	0.252	0.000 (−0.001–0.002)	0.388	0
Insulin resistance	0.018 (−0.018–0.055)	0.310	0.014 (−0.021–0.051)	0.412	0.004 (0.001–0.009)	0.017	22.2

Mediation structural equation model was adjusted for sex, age, time between exposure and outcome measure, high sensitivity C-reactive protein, heart rate, systolic blood pressure, smoking status, family history of hypertension/diabetes/high cholesterol/vascular disease, socioeconomic status, lean mass, high-density lipoprotein cholesterol, low-density lipoprotein cholesterol, triglyceride, glucose and insulin, sedentary time, light physical activity (light PA), or moderate-to-vigorous physical activity (MVPA) depending on the mediator. β is standardized regression co-efficient. *p*-value <0.05 were considered statistically significant.

## Data Availability

The informed consent obtained from ALSPAC participants does not allow the data to be made freely available through any third-party maintained public repository. However, data used for this submission can be made available on request to the ALSPAC Executive. The ALSPAC data management plan describes in detail the policy regarding data sharing, which is through a system of managed open access. Full instructions for applying for data access can be found here: http://www.bristol.ac.uk/alspac/researchers/access/. The ALSPAC study website contains details of all the data that are available (http://www.bristol.ac.uk/alspac/researchers/our-data/).
