# Supplementary material for: Arterial stiffness but not carotid intima-media thickness progression precedes premature structural and functional cardiac damage in youth: A 7-year temporal and mediation longitudinal study
Source: Atherosclerosis. Author manuscript; Available in PMC 2025 Jul 3. (PMC12225735; doi:10.1016/j.atherosclerosis.2023.117197)
Supplement: supplemental material [file NIHMS2083033-supplement-supplemental_material.pdf]

Supplemental Material

Arterial Stiffness but not Carotid Intima-Media Thickness Progression Precedes Premature Structural and Functional Cardiac Damage: A 7-year Temporal Causal and Mediation Longitudinal Study of 1856 Adolescents

Andrew O. Agbaje MD, MPH[1], Justin P. Zachariah MD, MPH[2], Tomi-Pekka Tuomainen MD, PhD[1]

[1]Institute of Public Health and Clinical Nutrition, School of Medicine, University of Eastern Finland, Kuopio, Finland; [2]Section of Pediatric Cardiology, Department of Pediatrics, Texas Children's Hospital, Baylor College of Medicine, Houston, Texas, United States.

Address correspondence to:
Andrew O. Agbaje, MD, MPH, Cert. Clinical Research (Harvard)
Institute of Public Health and Clinical Nutrition, School of Medicine, Faculty of Health Sciences, University of Eastern Finland, Kuopio Campus.
Address: Yliopistonranta 1, P.O. Box 1627, 70211 Kuopio, Finland
E-mail: andrew.agbaje@uef.fi

Supplemental Table 1 Percentile distribution of vascular and cardiac structure and function measures at baseline and follow-up.

	Left ventricular mass index$^{2.7}$ (g/m$^{2.7}$)				Relative wall thickness			
	17 years (baseline)		24 years (follow-up)		17 years (baseline)		24 years (follow-up)	
Percentiles	***Male***	***Female***	***Male***	***Female***	***Male***	***Female***	***Male***	***Female***
p10	27.74	26.15	30.46	27.63	0.32	0.32	0.30	0.29
p50	36.79	34.35	40.26	36.24	0.37	0.37	0.36	0.35
p75	42.73	39.14	45.76	41.82	0.41	0.40	0.40	0.39
p90	48.03	43.89	53.09	47.08	0.45	0.44	0.43	0.42
p95	51.63	47.27	56.84	50.13	0.49	0.49	0.45	0.44
Percentiles	**Left ventricular diastolic function (E/A)**				**Left ventricular filling pressure (E/e´)**			
p10	1.51	1.48	1.38	1.40	3.65	3.76	3.74	3.96
p50	1.89	1.86	1.91	1.88	4.74	4.78	4.66	5.08
p75	2.17	2.13	2.32	2.26	5.39	5.45	5.34	5.81
p90	2.47	2.40	2.65	2.76	6.15	6.12	6.18	6.51
p95	2.67	2.65	3.02	3.15	6.85	6.55	6.79	7.18
Percentiles	**Carotid-femoral pulse wave velocity (m/s)**				**Carotid intima-media thickness (mm)**			
p10	5.29	4.82	5.60	5.20	0.42	0.42	0.46	0.46
p50	5.99	5.53	6.53	5.97	0.48	0.47	0.52	0.52
p75	6.43	5.91	7.27	6.55	0.51	0.50	0.58	0.56
p90	6.85	6.26	8.28	7.50	0.54	0.53	0.64	0.62
p95	7.23	6.61	9.11	8.24	0.56	0.55	0.69	0.65

Supplemental Table 2 Effect of arterial stiffness and carotid intima-media thickness progression from ages 17 through 24 years on the risk of worsening left ventricular hypertrophy and diastolic dysfunction from ages 17 through 24 years according to sex.

Male n=845	**Worsening LVH**		**Increasing RWT**		**Worsening LVDD**		**Increasing LVFP**	
	Odds ratio (95% CI)	*p-value*	*Odds ratio (95% CI)*	*p-value*	*Odds ratio (95% CI)*	*p-value*	*Odds ratio (95% CI)*	*p-value*
**Carotid-femoral pulse wave velocity progression**								
Low category	Reference	–	Reference	–	Reference	–	Reference	–
Moderate category	0.91 (0.89 – 1.03)	0.126	0.96 (0.81 – 1.13)	0.594	0.97 (0.78 – 1.22)	0.820	1.11 (0.84 – 1.45)	0.477
High category	1.22 (1.08 – 1.38)	**0.002**	0.78 (0.70 – 0.91)	**0.002**	1.03 (0.92 – 1.14)	0.647	0.94 (0.56 – 1.56)	0.799
**Carotid intima-media thickness progression**								
Low category	Reference	–	Reference	–	Reference	–	Reference	–
Moderate category	1.04 (1.02 – 1.07)	**0.002**	1.03 (0.80 – 1.32)	0.819	0.88 (0.80 – 0.97)	**0.007**	0.97 (0.90 – 1.06)	0.507
High category	0.98 (0.91 – 1.05)	0.576	1.19 (0.84 – 1.69)	0.320	1.13 (0.94 – 1.37)	0.184	0.93 (0.92 – 0.94)	**<0.0001**
Female n=1011								
**Carotid-femoral pulse wave velocity progression**								
Low category	Reference	–	Reference	–	Reference	–	Reference	–
Moderate category	1.09 (1.06 – 1.13)	**<0.001**	1.11 (0.84 – 1.47)	0.481	1.21 (1.14 – 1.29)	**<0.001**	1.22 (1.04 – 1.43)	**0.016**
High category	1.11 (0.88 – 1.40)	0.367	0.85 (0.78 – 0.94)	**<0.001**	1.11 (0.94 – 1.30)	0.223	1.24 (1.22 – 1.26)	**<0.0001**
**Carotid intima-media thickness progression**								
Low category	Reference	–	Reference	–	Reference	–	Reference	–
Moderate category	0.97 (0.74 – 1.26)	0.808	0.82 (0.77 – 0.87)	**<0.001**	1.48 (1.43 – 1.54)	**<0.0001**	1.18 (1.13 – 1.24)	**<0.001**
High category	1.04 (0.87 – 1.24)	0.694	0.98 (0.67 – 1.44)	0.905	1.26 (0.68 – 2.33)	0.462	0.97 (0.97 – 0.98)	**<0.0001**

Multivariable analyses were adjusted for time in years between ages 17.7 and 24.5 years, age at baseline and other time varying covariates measured at both baseline and follow-up such as low-density lipoprotein cholesterol, insulin, triglyceride, high-sensitivity C reactive protein, high-density lipoprotein cholesterol, heart rate, glucose, systolic blood pressure, fat mass, lean mass, smoking status, family history of hypertension/diabetes/high cholesterol/vascular disease, social-economic status, and moderate to vigorous physical activity, light physical activity and sedentary time at 15 and 24 years.
Skewed predictors and covariates were logarithmically transformed before analyses. Odds ratio are follow-up time X predictor interaction effect computed using generalized logit mixed-effect model for repeated measures; CI, confidence interval; LVDD, left ventricular diastolic dysfunction; LVFP, left ventricular filling pressure; LVH, left ventricular hypertrophy; $LVMI^{2.7}$, left ventricular mass indexed for $height^{2.7}$. A 2-sided P-value <0.05 is considered statistically significant. Multiple testing was corrected with Sidak correction. Predictors were categorized in tertiles: tertile 1 is low, tertile 2 as moderate and tertile 3 as high categories. Participants with $LVMI^{2.7} \geq 51 g/m^{2.7}$, relative wall thickness ≥ 0.44, LVD function <1.5, and LVFP ≥ 8 were categorised as having LVH, increased RWT, LVDD and increased LVFP respectively. Multiple imputations were used to account for missing variables.

Supplemental Table 3 Effect of arterial stiffness and carotid intima-media thickness progression from ages 17 through 24 years on the risk of worsening left ventricular hypertrophy and diastolic dysfunction from ages 17 through 24 years according to systolic blood pressure category of normotensive and elevated blood pressure/hypertension at baseline.

Normotensive n=1745	**Worsening LVH**		**Increasing RWT**		**Worsening LVDD**		**Increasing LVFP**	
	Odds ratio (95% CI)	*p-value*	*Odds ratio (95% CI)*	*p-value*	*Odds ratio (95% CI)*	*p-value*	*Odds ratio (95% CI)*	*p-value*
**Carotid-femoral pulse wave velocity progression**								
Low category	Reference	–	Reference	–	Reference	–	Reference	–
Moderate category	0.97 (0.81 – 1.17)	0.782	0.90 (0.62 – 1.29)	0.551	1.17 (0.95 – 1.44)	0.145	0.87 (0.82 – 0.93)	**<0.001**
High category	1.16 (1.05 – 1.28)	**0.003**	0.93 (0.77 – 1.12)	0.430	1.07 (0.99 – 1.14)	0.073	0.94 (0.93 – 0.94)	**<0.0001**
**Carotid intima-media thickness progression**								
Low category	Reference	–	Reference	–	Reference	–	Reference	–
Moderate category	1.03 (0.79 – 1.36)	0.825	0.92 (0.81 – 1.04)	0.167	1.22 (1.20 – 1.24)	**<0.0001**	1.21 (0.95 – 1.53)	0.127
High category	1.07 (0.85 – 1.36)	0.571	1.14 (0.76 – 1.71)	0.517	1.11 (0.84 – 1.46)	0.480	1.15 (0.96 – 1.38)	0.136
Elevated systolic blood pressure / hypertension n=109								
**Carotid-femoral pulse wave velocity progression**								
Low category	Reference	–	Reference	–	Reference	–	Reference	–
Moderate category	0.76 (0.57 – 1.02)	0.067	0.78 (0.50 – 1.23)	0.292	0.73 (0.52 – 1.03)	0.075	0.86 (0.40 – 1.85)	0.702
High category	0.60 (0.55 – 0.65)	**<0.0001**	0.72 (0.69 – 0.75)	**<0.0001**	1.38 (0.46 – 4.13)	0.563	1.53 (0.68 – 3.41)	0.303
**Carotid intima-media thickness progression**								
Low category	Reference	–	Reference	–	Reference	–	Reference	–
Moderate category	0.97 (0.95 – 0.99)	**0.023**	1.20 (0.80 – 1.81)	0.383	0.79 (0.60 – 1.02)	0.077	1.13 (0.93 – 1.37)	0.222
High category	0.84 (0.69 – 1.01)	0.059	1.66 (1.11 – 2.49)	**0.014**	1.35 (0.82 – 2.25)	0.236	1.50 (1.22 – 1.84)	**<0.001**

Multivariable analyses were adjusted for sex, time in years between ages 17.7 and 24.5 years, age at baseline and other time varying covariates measured at both baseline and follow-up such as low-density lipoprotein cholesterol, insulin, triglyceride, high-sensitivity C reactive protein, high-density lipoprotein cholesterol, heart rate, glucose, lean mass, fat mass, smoking status, family history of hypertension/diabetes/high cholesterol/vascular disease, social-economic status, and moderate to vigorous physical activity, light physical activity and sedentary time at 15 and 24 years.
Skewed predictors and covariates were logarithmically transformed before analyses. Odds ratio are follow-up time X predictor interaction effect computed using generalized logit mixed-effect model for repeated measures; CI, confidence interval; LVDD, left ventricular diastolic dysfunction; LVFP, left ventricular filling pressure; LVH, left ventricular hypertrophy; $LVMI^{2.7}$, left ventricular mass indexed for $height^{2.7}$. A 2-sided P-value <0.05 is considered statistically significant. Multiple testing was corrected with Sidak correction. Predictors were categorized in tertiles: tertile 1 is low, tertile 2 as moderate and tertile 3 as high categories. Participants with $LVMI^{2.7}$ $\geq 51 g/m^{2.7}$, relative wall thickness ≥ 0.44, LVD function <1.5, and LVFP ≥ 8 were categorised as having LVH, increased RWT, LVDD and increased LVFP respectively. Multiple imputations were used to account for missing variables. Elevated systolic blood pressure/hypertension ≥130 mmHg.

Supplemental Table 4 Effect of arterial stiffness and carotid intima-media thickness progression from ages 17 through 24 years on the risk of worsening left ventricular hypertrophy and diastolic dysfunction from ages 17 through 24 years according to body mass index category of normal weight and overweight/obese at baseline.

Normal weight n=1455	**Worsening LVH**		**Increasing RWT**		**Worsening LVDD**		**Increasing LVFP**	
	Odds ratio (95% CI)	*p-value*	*Odds ratio (95% CI)*	*p-value*	*Odds ratio (95% CI)*	*p-value*	*Odds ratio (95% CI)*	*p-value*
**Carotid-femoral pulse wave velocity progression**								
Low category	Reference	–	Reference	–	Reference	–	Reference	–
Moderate category	0.92 (0.84 – 1.02)	0.104	0.97 (0.51 – 1.84)	0.923	1.18 (0.96 – 1.46)	0.108	0.92 (0.77 – 1.09)	0.323
High category	1.08 (0.97 – 1.21)	0.141	0.94 (0.85 – 1.04)	0.209	1.26 (1.09 – 1.47)	**0.002**	1.16 (1.02 – 1.32)	**0.026**
**Carotid intima-media thickness progression**								
Low category	Reference	–	Reference	–	Reference	–	Reference	–
Moderate category	1.01 (0.85 – 1.21)	0.886	0.81 (0.66 – 0.99)	**0.036**	1.09 (1.07 – 1.10)	**<0.0001**	1.06 (1.02 – 1.09)	**0.001**
High category	1.09 (0.96 – 1.24)	0.183	1.08 (0.89 – 1.31)	0.439	1.11 (0.87 – 1.42)	0.387	0.89 (0.82 – 0.95)	**0.002**
Overweight / obese n=401								
**Carotid-femoral pulse wave velocity progression**								
Low category	Reference	–	Reference	–	Reference	–	Reference	–
Moderate category	0.89 (0.76 – 1.04)	0.139	0.70 (0.65 – 0.76)	**<0.0001**	1.21 (0.94 – 1.54)	0.133	1.16 (1.03 – 1.32)	**0.018**
High category	1.06 (1.05 – 1.08)	**<0.001**	0.68 (0.59 – 0.80)	**<0.001**	0.81 (0.76 – 0.86)	**<0.001**	1.02 (0.93 – 1.11)	0.743
**Carotid intima-media thickness progression**								
Low category	Reference	–	Reference	–	Reference	–	Reference	–
Moderate category	1.18 (0.80 – 1.72)	0.404	0.93 (0.87 – 0.99)	**0.046**	1.24 (1.10 – 1.39)	**<0.001**	1.10 (1.08 – 1.12)	**<0.0001**
High category	1.08 (1.03 – 1.12)	**<0.001**	0.90 (0.80 – 1.01)	0.062	1.45 (0.87 – 2.40)	0.153	1.05 (0.93 – 1.18)	0.423

Multivariable analyses were adjusted for sex, time in years between ages 17.7 and 24.5 years, age at baseline and other time varying covariates measured at both baseline and follow-up such as low-density lipoprotein cholesterol, insulin, triglyceride, high-sensitivity C reactive protein, high-density lipoprotein cholesterol, heart rate, glucose, systolic blood pressure, smoking status, family history of hypertension/diabetes/high cholesterol/vascular disease, social-economic status, and moderate to vigorous physical activity, light physical activity and sedentary time at 15 and 24 years.

Skewed predictors and covariates were logarithmically transformed before analyses. Odds ratio are follow-up time X predictor interaction effect computed using generalized logit mixed-effect model for repeated measures; CI, confidence interval; LVDD, left ventricular diastolic dysfunction; LVFP, left ventricular filling pressure; LVH, left ventricular hypertrophy; $LVMI^{2.7}$, left ventricular mass indexed for $height^{2.7}$. A 2-sided P-value <0.05 is considered statistically significant. Multiple testing was corrected with Sidak correction. Predictors were categorized in tertiles: tertile 1 is low, tertile 2 as moderate and tertile 3 as high categories. Participants with $LVMI^{2.7} \geq 51 g/m^{2.7}$, relative wall thickness ≥ 0.44, LVD function <1.5, and LVFP ≥ 8 were categorised as having LVH, increased RWT, LVDD and increased LVFP respectively. Multiple imputations were used to account for missing variables.

Supplemental Table 5 Effect of arterial stiffness and carotid intima-media thickness progression from ages 17 through 24 years on the risk of worsening left ventricular hypertrophy and diastolic dysfunction from ages 17 through 24 years with complete predictor and outcome variables at baseline and follow-up

N=839	Worsening LVH		Increasing RWT		Worsening LVDD		Increasing LVFP	
	Odds ratio (95% CI)	*p-value*	*Odds ratio (95% CI)*	*p-value*	*Odds ratio (95% CI)*	*p-value*	*Odds ratio (95% CI)*	*p-value*
**Carotid-femoral pulse wave velocity progression**								
Low category	Reference	–	Reference	–	Reference	–	Reference	–
Moderate category	1.15 (0.39 – 3.35)	0.802	0.55 (0.29 – 1.07)	**<0.0001**	0.944 (0.68 – 1.32)	0.735	0.74 (0.73 – 0.76)	**<0.0001**
High category	1.85 (1.70 – 2.01)	**<0.0001**	0.37 (0.35 – 0.38)	0.076	2.00 (1.15 – 3.49)	**0.014**	0.92 (0.91 – 0.94)	**<0.001**
**Carotid intima-media thickness progression**								
Low category	Reference	–	Reference	–	Reference	–	Reference	–
Moderate category	1.53 (0.62 – 3.79)	0.357	1.75 (1.10 – 2.79)	**0.019**	1.25 (0.93 – 1.68)	0.135	0.95 (0.87 – 1.04)	0.267
High category	0.75 (0.19 – 3.07)	0.692	1.28 (0.79 – 2.07)	0.959	1.90 (0.64 – 5.59)	0.252	0.82 (0.75 – 0.89)	**<0.001**

Multivariable analyses were adjusted for sex, time in years between ages 17.7 and 24.5 years, age at baseline and other time varying covariates measured at both baseline and follow-up such as low-density lipoprotein cholesterol, insulin, triglyceride, high-sensitivity C reactive protein, high-density lipoprotein cholesterol, heart rate, glucose, systolic blood pressure, fat mass, lean mass, smoking status, family history of hypertension/diabetes/high cholesterol/vascular disease, social-economic status, and moderate to vigorous physical activity, light physical activity and sedentary time at 15 and 24 years.

Skewed predictors and covariates were logarithmically transformed before analyses. Odds ratio are follow-up time X predictor interaction effect computed using generalized logit mixed-effect model for repeated measures; CI, confidence interval; LVDD, left ventricular diastolic dysfunction; LVFP, left ventricular filling pressure; LVH, left ventricular hypertrophy; $LVMI^{2.7}$, left ventricular mass indexed for $height^{2.7}$. A 2-sided P-value <0.05 is considered statistically significant. Multiple testing was corrected with Sidak correction. Predictors were categorized in tertiles: tertile 1 is low, tertile 2 as moderate and tertile 3 as high categories. Participants with $LVMI^{2.7} \geq 51 g/m^{2.7}$, relative wall thickness ≥ 0.44, LVD function <1.5, and LVFP ≥ 8 were categorised as having LVH, increased RWT, LVDD and increased LVFP respectively. Multiple imputations were used to account for missing covariates.

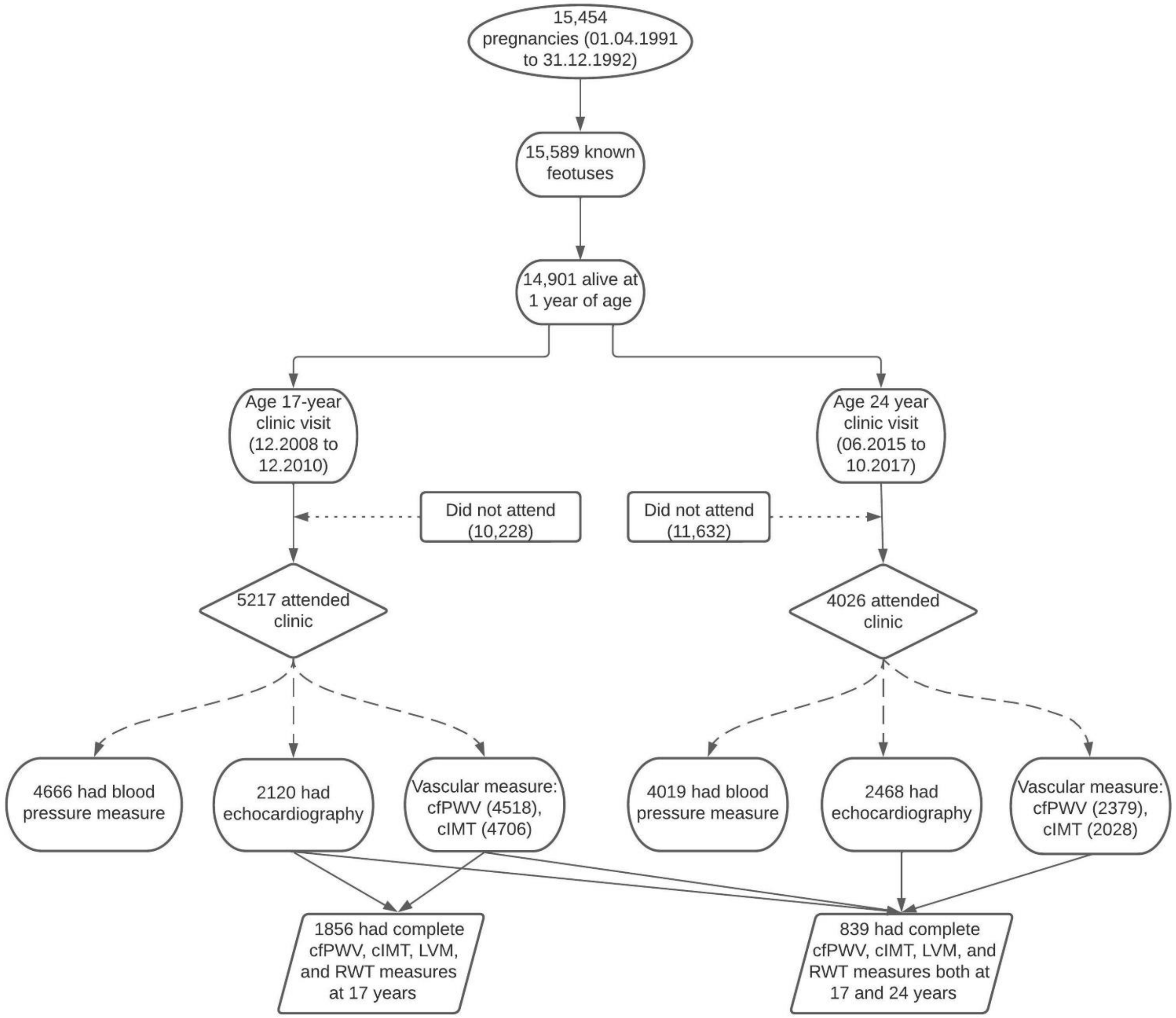
15,454 pregnancies (01.04.1991 to 31.12.1992)
15,589 known feotuses
14,901 alive at 1 year of age
Age 17-year clinic visit (12.2008 to 12.2010)
Age 24 year clinic visit (06.2015 to 10.2017)
Did not attend (10,228)
Did not attend (11,632)
5217 attended clinic
4026 attended clinic
4666 had blood pressure measure
2120 had echocardiography
Vascular measure: cfPWV (4518), cIMT (4706)
4019 had blood pressure measure
2468 had echocardiography
Vascular measure: cfPWV (2379), cIMT (2028)
1856 had complete cfPWV, cIMT, LVM, and RWT measures at 17 years
839 had complete cfPWV, cIMT, LVM, and RWT measures both at 17 and 24 years

Supplemental Figure 1 Flowchart of study participants

cfPWV, carotid-femoral pulse wave velocity; cIMT, carotid intima-media thickness; LVM, left ventricular mass; RWT, relative wall thickness. Participants that had complete predictor and or outcome of interest at 17 years were included in the analyses.
